# The Association between the Self-Management of Mild Symptoms and Quality of Life of Elderly Populations in Rural Communities: A Cross-Sectional Study

**DOI:** 10.3390/ijerph18168857

**Published:** 2021-08-23

**Authors:** Ryuichi Ohta, Mikiya Sato, Jun Kitayuguchi, Tetsuhiro Maeno, Chiaki Sano

**Affiliations:** 1 Community Care, Unnan City Hospital, Unnan, 96-1 Iida, Daito-cho, Unnan 699-1221, Japan; 2 Department of Primary Care and Medical Education, Graduate School of Comprehensive Human Sciences, University of Tsukuba, Tsukuba 305-8575, Japan; 3 Faculty of Medicine, University of Tsukuba, Tsukuba 305-8575, Japan; mikiya.sato@shi-g.com (M.S.); maenote@md.tsukuba.ac.jp (T.M.); 4 Health Services Center, Occupational Safety and Health Department, Human Resources Group, Sumitomo Heavy Industries, Ltd., Tokyo 141-6025, Japan; 5 Physical Education and Medicine Research Center Unnan, Unnan 699-1105, Japan; junk_907@yahoo.co.jp; 6 Department of Community Medicine Management, Faculty of Medicine, Shimane University, 89-1 Enya cho, Izumo 693-8501, Japan; sanochi@med.shimane-u.ac.jp

**Keywords:** help-seeking behavior, self-management, self-care, quality of life, rural community, EQ-5D-5L, Japan

## Abstract

Maintaining people’s health based on their help-seeking behavior (HSB) regarding mild symptoms is essential. An effective HSB, especially self-management, can facilitate the attainment of appropriate healthcare resources and affect health outcomes such as quality of life (QOL). However, clear evidence regarding the relationship between self-management, mild symptoms, and QOL is unavailable. Therefore, this cross-sectional study investigated this association in a rural elderly population. The participants, aged over 65 years, were living in rural communities. The primary outcome of QOL was examined using the EuroQol 5-Dimension 5-Level (EQ-5D-5L). After adjusting for propensity score matching, 298 participants in the self-management usage group were matched with 298 in the group not using self-management. The most frequent HSB trend was consulting with primary care physicians, followed by self-care, consulting with families, utilizing home medicines, and buying medicines. The EQ-5D-5L scores were statistically higher in the self-management usage group than in the other group. The HSBs with a trend of using self-management were related to a high QOL. Self-management of symptoms along with other HSBs can improve elderly HSBs in rural contexts. Educational interventions and system development for HSBs in rural contexts could be effective in enhancing the QOL of rural elderly populations.

## 1. Introduction

An increase in the world’s aging population leads to increasing health problems among the elderly, particularly in developed countries, that demand complicated health management [1]. The elderly suffer from several age-related diseases that require adequate management through established healthcare systems [2]. More than 70% of adults over 65 suffer from chronic diseases such as hypertension, diabetes, heart diseases, cancer, and cerebrovascular diseases, accounting for 60% of deaths. The growing number of ailments and frequency of visiting medical institutions increase medical expenses, thus causing financial challenges [3]. To avoid illnesses and their progression, primary and tertiary prevention is crucial for ensuring only mild conditions among the elderly [4]. Help-seeking behaviors (HSB) must be modified and improved from a health perspective to facilitate disease prevention. HSBs are human behaviors for maintaining health and seeking treatment for abnormal health symptoms [5]. HSBs are essential for managing citizens’ health conditions and are exhibited when an individual experiences disease-related difficulty. Furthermore, each citizen should have appropriate HSBs to maintain their health [6]. For appropriate HSBs, a balance between lay and professional care is important [7]. Lay care refers to the unpaid care provided by amateurs with no formal training, such as self-management and care by relatives, friends, and self-help groups [3]. Professional care is provided by trained, paid professionals, usually in a formal setting [3]. Using both types of care can efficiently reduce inappropriate HSBs to treat mild symptoms [8]. Furthermore, elderly populations’ HSBs, especially regarding self-management, must be strengthened [9]. Over 70% of the elderly have chronic diseases with a high potential of severe complications [10]. Rural older adults tend to keep to themselves or visit medical institutions without consulting others [11]. Among the rural Japanese elderly, 65.8% use professional care monthly compared to 20.6% in urban areas [12]. As rural older adults depend on professional care, adequate HSBs for minor symptoms can moderate their progression through early detection, especially in rural contexts.

HSBs are influenced by various factors, including biological, social, economic, and cultural factors. Additionally, the disease burden alters HSBs, engendering frequent visits to medical institutions [13]. Isolation and poor socioeconomic conditions can impair HSBs through high anxiety and low health literacy [14]. Furthermore, HSBs are also influenced by nationality and demographics. In rural areas, there is a dearth of healthcare professionals and individuals who are knowledgeable about HSBs [15,16]. The rural elderly’s HSBs are affected by their health literacy. Based on previous studies, the elderly tend to be anxious about their understanding of symptoms and exhibit inappropriate HSBs in their lives [16]. Moreover, Japan’s culture impacts HSBs because Japanese health insurance supports various medical tests and procedures; many older individuals perform prompt routine visits to medical institutions despite only having mild symptoms [17]. HSBs can be affected by health conditions and their traits, thus causing healthcare problems; an inappropriate HSB can impinge on health outcomes such as self-rated health (SRH) and quality of life (QOL) [18,19], which can be affected by an individual’s perception of their living environment. Effective usage of health care and resources in their environment may improve their QOL [18,19]. A previous study conducted in rural areas showed that individuals’ HSBs for mild symptoms in which both lay and professional care were used were associated with improved SRH.

Although clear evidence regarding the association between the elderly’s HSBs and QOL is unavailable, its clarification could be critical for managing the HSBs and health conditions of older adults. Rural areas require appropriate HSBs for the elderly, especially regarding self-management, to ensure sustainable healthcare [20]. Self-management can be associated with older adults’ motivation to be in control of their health, which can be related to QOL [20]. In rural areas, not only are healthcare resources declining but healthcare professionals are also aging [21,22]. As a vast number of people live in rural areas worldwide, investigating the issues of HSBs in these areas is beneficial for global healthcare [23]. By elucidating the relationship between self-management and QOL, the adequate usage of healthcare resources and appropriate HSBs, including self-management, could be enhanced. Moreover, education could be provided to rural communities through a systems approach involving top–down and upstream interventions. Over 70% of the population experiences various mild symptoms in a single month that could be resolved through HSBs [24,25]. Thus, an investigation of the self-management of minor symptoms can be rewarding. As a previous study reported the usage of HSBs regarding mild symptoms, the relationship between HSB usage and QOL should be investigated. Thus, this study aimed to investigate the relationship between self-management of mild symptoms and QOL among the elderly in rural communities.

## 2. Materials and Methods

### 2.1. Setting

This research was performed in Kakeya and Yoshida in Unnan City, located in southeast Shimane prefecture, Japan; the area is mostly covered by forests and is one of the most rural cities in Japan. A 2017 survey revealed that the total population of Unnan City was 38,882 (18,720 males and 20,162 females), with an elderly rate of 37.82% that is estimated to reach 50% by 2025. Each family tended to live separately. Kakeya and Yoshida towns are located in the most southwestern part of Unnan City, where there are six regions in total. Participants from four regions (Kakeya, Tai, Matsukasa, and Tane) participated in this study.

### 2.2. Participants

Overall, 572, 247, 129, and 211 people over 65 years old resided in Kakeya, Tai Matsukasa, and Tane, respectively. These regions are neighboring districts. The participants were recruited from this population (i.e., over 65 years old living in these regions). They were informed about this study by a letter with an explanation and a research questionnaire. Those patients who could not read or write appropriately and those with dementia were excluded from this study.

### 2.3. Measurements

A questionnaire was distributed to all participants. Community workers in each region distributed the questionnaire to the participants and collected them. As a primary outcome, QOL was measured using EuroQol 5-Dimension 5-Level (EQ-5D-5L) that included five domains (mobility, self-care, usual activities, pain/discomfort, and anxiety/depression); it has been validated in various languages, including Japanese [26]. The Japanese version was used in this study. SRH was measured as a secondary outcome [27] by the question: “In general, would you say your health is good, relatively good, relatively bad, or bad?” These responses were dichotomized into poor (self-rating of relatively bad or bad) or good (self-rating of good or relatively good). To assess the patients’ application of HSBs for mild symptoms, a validated questionnaire was used [28]. In this questionnaire, the participants indicated their behaviors when they had mild symptoms. The choices included doing nothing, self-management (observing, sleeping, resting, and taking a bath for their symptoms), seeking information, consulting family and friends, consulting community members (people living nearby and members of the community center), using complementary medicine, consuming home medicines, buying over-the-counter drugs, consulting pharmacists, consulting primary care physicians, visiting medical institutions (other than primary care physicians), and visiting the emergency rooms of general hospitals (including calling an ambulance). Items assessing age, sex, body mass index (BMI), smoking, habitual alcohol consumption, educational level, living conditions, social support [29], social capital (using a 10-point Likert scale ranging from “can rely on neighbors in communities” to “cannot completely rely on neighbors in communities”) [30], and socioeconomic status (SES) were included in the questionnaire. The independent variables were categorized binomially: sex (male = 1, female = 0), smoking (yes = 1, no = 0), habitual alcohol drinking (yes = 1, no = 0), educational level (more than graduation from high school = 1, no = 0), living condition (with family = 1, residing alone = 0), SRH (good or relatively good = 1, relatively bad or bad = 0), social support (present or relatively present = 1, absent or relatively absent = 0), social capital (high (10 to 6) = 1, low (5 to 1) = 0), and socioeconomic status (high (rich or relatively rich) = 1, low (poor or relatively poor) = 0).

### 2.4. Statistical Analysis

The parametric and categorical data were analyzed using Students’ *t*-test and chi-squared test, respectively. A significance level of *p* < 0.05 was used for all comparisons. The participants were divided into two groups based on whether they practiced self-management (exposure and control). To investigate the statistical difference in their EQ-5D-5L scores, a minimum of 126 participants was required in each group based on α (alpha) = 0.05, β (beta) = 0.20, and a power of 80%. We employed a propensity score matching to adjust for the differences in demographic data between the two groups. The propensity scores were calculated using this study’s demographic factors. One-to-one caliper (0.2) matching was utilized with no replacement for the matching of the exposure and control groups. Additionally, the covariate balance between the matched groups was examined. All statistical analyses were performed using the Easy R (Saitama Medical Center, Jichi Medical University, Saitama, Japan), a graphical user interface for R (The R Foundation, Vienna, Austria) [31].

### 2.5. Ethical Considerations

The participants were informed that the data collected in this study would be used only for research purposes. Furthermore, they were informed about the research aims, how their data would be disclosed, and the protection of their personal information; subsequently, they provided written consent. The study was conducted in accordance with the principles of the Declaration of Helsinki. Furthermore, it was approved by the Unnan City Hospital Clinical Ethics Committee (approval number: 20200013).

## 3. Results

### 3.1. The Demographic Data of the Participants

**Figure 1:**
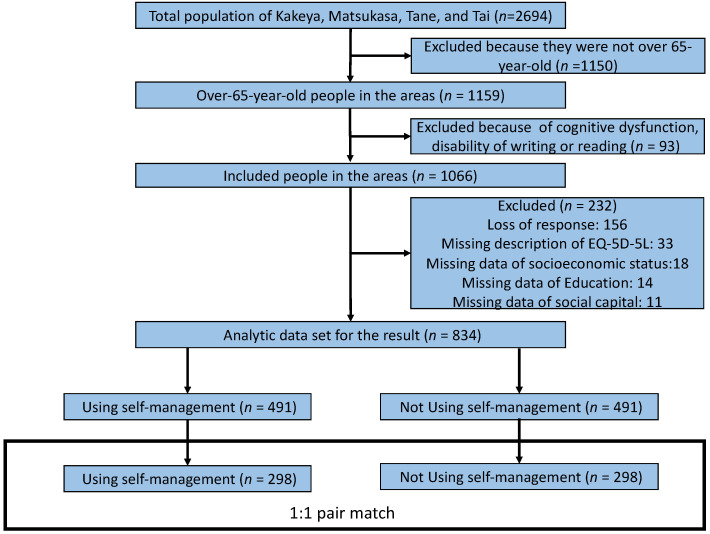
Flowchart of the participant selection process.

Figure 1 shows a flowchart of the participant selection process. The total population of Kakeya, Matsukasa, Tane, and Tai was 2694. Among them, 1159 were over 65 years old. We included a total of 1066 participants; of them, 232 were excluded due to the lack of responses and missing data regarding the description of EQ-5D-5L, socioeconomic status, education, and social capital (Figure 1).

**Figure 2:**
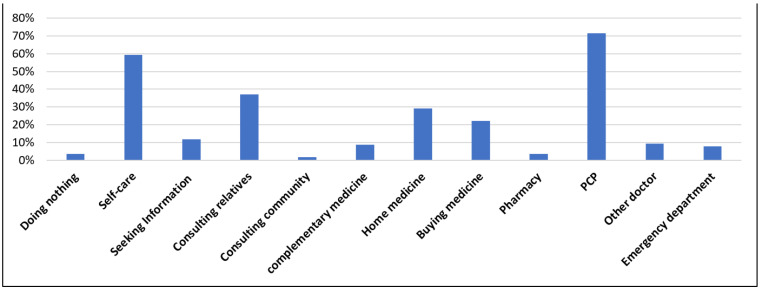
The percentage of each HSB regarding mild symptoms. Note: consulting relatives, consulting family and friends; consulting community, consulting community members; complementary medicine, using complementary medicine; home medicine, using home medicine; buying medicine, purchasing over-the-counter drugs; pharmacy, consulting with pharmacists; PCP, consulting with primary care physicians; other doctor, visiting medical institutions (other than primary care physicians); emergency department, visiting emergency rooms of general hospitals (including calling an ambulance).

The total effective response rate of the questionnaires was 78.2% (834/1066), and that of the participants from Kakeya, Tai, Matsukasa, and Tane was 80% (443/554), 67.9% (155/230), 97.2% (105/109), and 75.7% (131/173), respectively. The participants in the self-management group were younger than those in the non-self-management group (*p* = 0.003). Fewer participants in the former group had chronic diseases compared to those in the latter group (*p* < 0.001). More participants in the self-management group had higher education compared to the other group (*p* = 0.001). Additionally, a greater number of participants in the former had a higher SES and social support than the latter (*p* = 0.007 and 0.026, respectively). No significant differences were found in conditions regarding sex, BMI, alcohol use, tobacco use, living with family, annual health check-ups, and social capital between the groups (Table 1). After adjusting for the propensity score matching, 298 participants in the intervention group were matched with 298 participants in the control group. The C-statistic for the propensity score regression models was 0.629 (95% confidence interval [CI], 0.59–0.668). The most frequent HSB exhibited was consulting with primary care physicians (71.6%), followed by self-management (59.2%), consulting with families (37.1%), using home medicines (29.0%), and buying medicines (22.1%). The rates of consulting with community members (15.6%) and information gathering (11.8%) were low (Figure 2).

### 3.2. The Relationship between Uss of Self-Management, QOL, and SRH

Regarding the post propensity score matching, the scores of the EQ-5D-5L were statistically higher in the self-management group than in the non-self-management group (0.013). In the EQ-5D-5L, the scores of the subscales “QOL1: mobility” (*p* = 0.005), “QOL3: usual activities” (*p* = 0.217), and “QOL5: mobility” (*p* = 0.056) were greater in the former than in the latter. The subscales “QOL2: self-care” (*p* = 0.094) and “QOL4: pain/discomfort” (*p* = 0.289) were not significantly different between the groups. The SRH rate was higher in the self-management group than in the non-self-management group (Table 2).

## 4. Discussion

This study is the first to show that the behavioral trend of self-management with respect to mild symptoms is associated with a high QOL and SRH among the rural elderly. Frequently occurring HSBs included consulting with primary care physicians, self-management, usage of home medicines, and buying over-the-counter drugs; however, the rates of consultations with community members and information gathering were low. In rural contexts where healthcare resources are lacking, rural older adults’ use of HSBs should be managed effectively, with a focus on improving their ties with community members, knowledge of medicines, and revision of providing healthcare; this could facilitate the contribution of HSBs to their QOL.

In this study, the usage of self-management for mild symptoms was positively associated with QOL. Self-management entailed understanding their health conditions, such as pain and fatigue, and adjusting their behaviors accordingly [32,33]. An adjustment in HSBs may require people to observe and control their symptoms without dismissing the alarming ones [34,35,36]. Regulating HSBs can moderate body functions related to the continuity of daily life activities [11,12,13,37], which is one of the factors indicating higher QOL. As this study reported a relatively high difference between the two groups’ EQ-5D-5L scores in component 2 of self-care, individuals that utilize self-management for mild symptoms may tend to consider not only usual self-management but also changes in their health conditions in their approach to managing common health issues. In addition, their control methods could be modified by taking advice from their families and primary care physicians [6,38]. Furthermore, their suggestions could also help in the individuals’ usage of home medicines, along with the latter’s own knowledge and understanding of them [5,12]. The utilization of home medicines can be facilitated by families, community members, and healthcare professionals [39,40] that may be associated with daily life management and low anxiety, thus referring to components 3 and 5 of the EQ-5D-5L. Furthermore, this study indicated that the self-management of mild symptoms could be related to components 1 and 4 of mobility and less pain/discomfort, respectively. The former is associated with body functions and mental conditions that may be affected by pain or discomfort [41,42]. Older adults who perform self-management of their mild symptoms can show improved recognition in their mental and physical conditions, as this study reported them as having an increased SRH. Based on previous research, SRH can be associated with better health conditions and management [43]. Thus, components 1 and 4 could be high among such individuals. Although, in this research, we were unable to demonstrate the cause-and-effect relationship between the usage of lay care and QOL, enhancement of the former, supported by systems and education in communities, could improve QOL in rural older people.

For supporting systems of lay care in communities, a high and low rate of consultations with primary care physicians, as well as community members and other professionals, respectively, is crucial. Based on previous studies, primary care physicians are the first step in medical care and can facilitate the management of symptoms by modifying behaviors [44,45]. In rural settings, people have increased reliance on primary care physicians, whose advice is useful and critical [13,14]. As this study indicated a high usage of primary care physicians, their effective guidance on self-management could lead to its enhancement among the rural elderly, which should be promoted. In addition, the low utilization rate of community members could be significant for the continuity of rural communities [46]. Prior research has shown that these communities are aging rapidly and losing previously active relationships because of young people settling outside and a mixing of cultures [14,20,47]. Their mutual relationships can sustain their lives and solve each issue through collaborations [48]. Furthermore, the low rate of consultations with community members can also be related to the issues of privacy that may render rural individuals reluctant to confessing their difficulties to others [14]. As rural areas lack adequate healthcare and human resources, their present rural resources should be integrated efficiently. For the modification of HSBs, lay and professional resources need to be appropriately acknowledged and employed based on the needs of rural people.

Improvements in HSBs may require a continuous provision of information and revision of the healthcare system. In this study, the usage of HSBs, such as consulting with community members and information gathering, were low. For improved health behaviors, the engagement and empowerment of people are essential and should be cultivated by all community members [46,49]. To enhance the understanding, self-efficacy, and intention of performing appropriate HSBs, continual provision of information and dialogues among the rural elderly, community members, and healthcare professionals are needed [50]. Through these dialogues, the elderly can understand the present conditions of their knowledge of HSBs and healthcare resources, as well as become empowered and motivated to learn them with the help of health care professionals; this could contribute to the establishment of systems and education regarding HSBs with respect to their contexts.

This study has certain limitations. Due to its cross-sectional design, we could not demonstrate the cause-and-effect relationship between HSBs and QOL. Longitudinal studies are recommended to overcome this problem. As this research was conducted in Japanese super-aged rural communities, our sample had a selection bias. An investigation of a broader range of communities could offer an additionally nuanced understanding. Finally, this study’s sample size was relatively small, and a larger one with a wider range of demographics could address this limitation.

## 5. Conclusions

HSBs involving self-management were reported to be associated with a high QOL. Self-management with the practical use of other resources could be important for increasing HSBs. Educational interventions and the development of systems for HSBs in rural contexts could be effective in improving the rural elderly’s QOL. Furthermore, Japan is an aging country, and many developing and developed economies will follow this trend shortly. Our findings could be helpful for such communities.

## Figures and Tables

**Table 1:** Demographic data of the participants in each group and the significance level of the comparison among the two groups before and after propensity score matching.

	Before Propensity Score Weighting			After Propensity Score Matching		
	Using Self-Management	Not Using Self-Management	*p*-Value	Using Self-Management	Not Using Self-Management	*p*-Value
Variables	*n* = 491	*n* = 343		*n* = 298	*n* = 298	
Age (in years), mean (SD)	76.91 (7.85)	78.58 (8.14)	0.003	77.88 (8.17)	78.36 (8.09)	0.472
Sex, male (%) (Reference: female)	202 (41.2)	164 (47.8)	0.065	130 (43.6)	132 (44.3)	0.934
BMI, mean (SD)	22.72 (3.71)	22.51 (3.90)	0.436	22.65 (3.12)	22.54 (3.93)	0.708
Having chronic diseases (%) (Reference: no chronic diseases)	407 (82.9)	314 (91.5)	<0.001	271 (90.9)	271 (90.9)	1
Alcohol (%) (Reference: no alcohol)	175 (35.9)	109 (32.0)	0.265	96 (32.2)	92 (30.9)	0.791
Tobacco (%) (Reference: no smoking)	29 (5.9)	33 (9.6)	0.057	23 (7.7)	18 (6.0)	0.518
Higher Education (%) (Reference: lower education)	243 (49.8)	129 (37.9)	0.001	124 (41.6)	121 (40.6)	0.868
Living with family (%) (Reference: living alone)	425 (87.1)	296 (88.4)	0.667	263 (88.3)	262 (87.9)	1
Annual health check-up (%) (Reference: no annual health check-up)	358 (73.2)	238 (70.0)	0.346	208 (69.8)	213 (71.5)	0.719
High SES (%) (Reference: low SES)	280 (57.4)	160 (47.6)	0.007	150 (50.3)	151 (50.7)	1
High social capital (%) (Reference: low social capital)	415 (85.9)	277 (82.4)	0.202	253 (84.9)	252 (84.6)	1
High social support (%) (Reference: no alcohol)	422 (86.7)	275 (80.9)	0.026	249 (83.6)	248 (83.2)	1

**Table 2:** The participants in each group and the significance level of the comparison between the two groups before and after propensity score matching.

	Before Propensity Score Weighting			After Propensity Score Weighting		
	The Usage of Self-Management	No Use	*p*-Value	The Usage of Self-Management	No Use	*p*-Value
Variables	*n* = 491	*n* = 343		*n* = 298	*n* = 298	
EQ-5D-5L, mean (SD)	0.72 (0.17)	0.65 (0.23)	<0.001	0.70 (0.18)	0.66 (0.23)	0.013
QOL1: mobility	1.64 (1.01)	2.08 (1.34)	<0.001	1.75 (1.08)	2.03 (1.33)	0.005
QOL2: self-care	1.36 (0.81)	1.66 (1.13)	<0.001	1.46 (0.94)	1.60 (1.10)	0.094
QOL3: usual activities	1.69 (0.91)	2.04 (1.15)	<0.001	1.76 (0.96)	1.97 (1.12)	0.017
QOL4: pain/discomfort	2.19 (0.88)	2.38 (1.05)	0.004	2.26 (0.90)	2.34 (1.03)	0.289
QOL5: anxiety/depression	1.70 (0.81)	1.95 (0.97)	<0.001	1.75 (0.85)	1.89 (0.95)	0.056
high SRH (%)	380 (77.4)	217 (63.3)	<0.001	221 (74.2)	198 (66.4)	0.048

## Data Availability

The datasets used and/or analyzed during the current study may be obtained from the corresponding author upon reasonable request.
